# Supplementary material for: A neuronal basis for fear discrimination in the lateral amygdala
Source: Nat Commun. 2018 Mar 23;9:1214. doi: 10.1038/s41467-018-03682-2 (PMC5865209; doi:10.1038/s41467-018-03682-2)
Supplement: Supplementary file 1 — Supplementary Information(PDF 1288 kb) [file 41467_2018_3682_MOESM1_ESM.pdf]

A neuronal basis for fear discrimination in the lateral amygdala

Authors: Anna Grosso, Giulia Santoni, Eugenio Manassero, Annamaria Renna and Benedetto Sacchetti

Supplementary Information

Supplementary Note 1

Figure 4

(**e**) New Tone (7 kHz, pre-injection) and Tone (7 kHz, post-injection): A 3×2 mixed ANOVA model showed a significant main effect of group ($F_{(2, 19)} = 7.25$, $P < 0.01$), a significant main effect of trial ($F_{(1, 19)} = 9.23$, $P < 0.01$) and a significant group×trial interaction ($F_{(2, 19)} = 20.07$, $P < 0.001$). Simple main effects analysis indicated a significant difference between groups in post-injection trial ($F_{(2, 19)} = 24.13$, $P < 0.001$) but not in pre-injection trial ($F_{(2, 19)} = 0.90$, $P > 0.05$). In post-injection trial, freezing levels of Daun02-injected rats were significantly higher than freezing levels in the other two conditions ($P < 0.001$ in both cases). CS (1 kHz, post-injection): A one-way ANOVA indicated no significant differences between groups ($F_{(2, 19)} = 0.24$, $P > 0.05$).

(**h**) Tones 1-7 (Extinction session - Day 1): A 2×7 mixed ANOVA yielded a not significant main effect of group ($F_{(1, 10)} = 0.31$, $P > 0.05$) and a not significant group×trial interaction ($F_{(2.70, 26.99)} = 0.21$, $P > 0.05$). The main effect of trial was significant ($F_{(2.70, 26.99)} = 3.13$, $P < 0.05$), indicating a general decrease in freezing response levels. Tones 30-36 (Extinction session - Day 1): A 2×7 mixed ANOVA model showed a not significant main effect of group ($F_{(1, 10)} = 4.01$, $P > 0.05$), a not significant main effect of trial ($F_{(6, 60)} = 0.33$, $P > 0.05$) and a not significant group×trial interaction ($F_{(6, 60)} = 0.21$, $P > 0.05$). Tones 30-36 (Extinction session - Day 2): A 2×7 mixed ANOVA model showed a not significant main effect of group ($F_{(1, 10)} = 0.004$, $P > 0.05$), a not significant main effect of trial ($F_{(2.90, 29.03)} = 1.30$, $P > 0.05$) and a not significant group×trial interaction ($F_{(2.90, 29.03)} = 1.44$, $P > 0.05$).

(**i**) CS (1 kHz, pre-injection) and CS (1 kHz, post-injection): A 3×2 mixed ANOVA revealed a significant main effect of group ($F_{(2, 13)} = 15.36$, $P < 0.001$) and a significant group×trial interaction ($F_{(2, 13)} = 14.11$, $P = 0.001$). The main effect of trial was not significant ($F_{(1, 13)} = 0.80$, $P > 0.05$). Simple main effects analysis indicated a significant difference between groups in post-injection trial ($F_{(2, 13)} = 20.57$, $P < 0.001$) but not in pre-injection trial ($F_{(2, 13)} = 2.83$, $P > 0.05$). In post-injection trial, freezing levels of Daun02-injected rats were significantly lower than freezing levels in the other two

conditions (P < 0.001 in both cases). New Tone (7 kHz, post-injection): A one-way ANOVA indicated no significant differences between groups ($F_{(2, 13)} = 0.99$, $P > 0.05$).

(**j**) New Tone (15 kHz, pre-injection) and Tone (15 kHz, post-injection): A 2×2 mixed ANOVA model showed a not significant main effect of group ($F_{(1, 16)} = 0.74$, $P > 0.05$), a not significant main effect of trial ($F_{(1, 16)} = 0.01$, $P > 0.05$) and a not significant group×trial interaction ($F_{(1, 16)} = 1.75$, $P > 0.05$).

Supplementary Figures and Legends

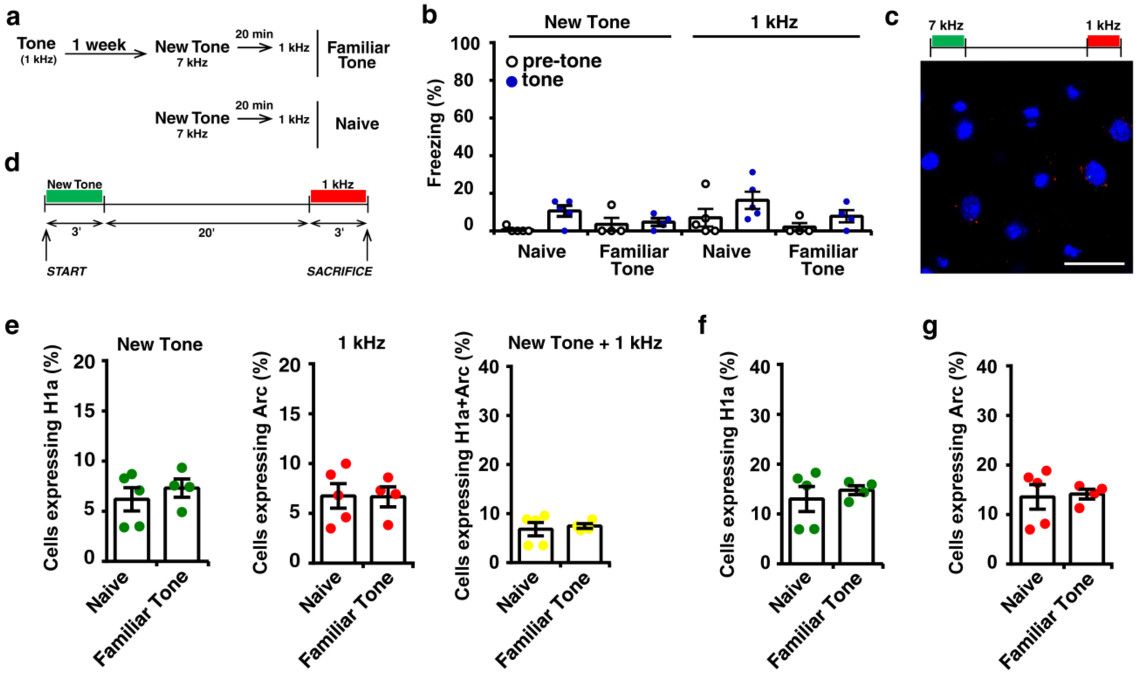

Supplementary Figure 1. LA activity in naïve and familiar tone groups following the presentation of 7 kHz and 1 kHz tones. (a) Experimental design of behavioral and catFISH experiments of familiar tone group and totally naïve group. In the familiar tone group, during training rats were presented with the 1 kHz tone unaccompanied by any painful stimulation. One week later, rats were presented with a new tone (7 kHz) followed 20 min later by the 1 kHz tone. Totally naive animals were presented with the two tones only during the test trial. **(b)** The percentage of freezing during 7 kHz tone presentation was similar between naive and familiar tone groups ($t_{(7)}$= 1.59, P > 0.05). Similar results were obtained during the presentation of the 1 kHz tone ($t_{(7)}$= 1.43, P > 0.05). **(c)** Representative image showing *H1a*- and *Arc*-expressing neurons in the familiar tone group (*n* = 4). (**d**) Experimental design of catfish experiment. **(e)** The percentages of neurons expressing *H1a* ($t_{(7)}$= -0.73, P > 0.05), *Arc* ($t_{(7)}$= 0.05, P > 0.05) or both ($t_{(7)}$= -0.41, P > 0.05) were similar in the naive and familiar tone groups. **(f,**

g) No differences were detected between groups in the total rate of *H1a* ($t_{(7)}$= -0.6, $P > 0.05$)and in the Arc total rate($t_{(7)}$= -0.19, $P > 0.05$). All data are mean and SEM.

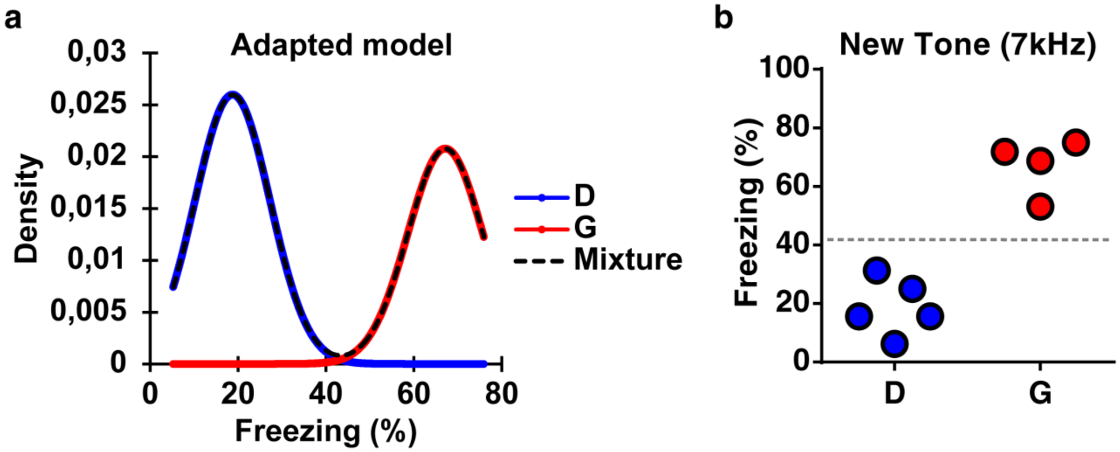

Supplementary Figure 2. Cluster analysis of behavioral response to the New Tone. (**a**) Two-component Gaussian mixture model (GMM) showing the distribution of discriminator (blue) and generalizer (red) animals presented with the New 7 kHz tone. The threshold estimated through the expectation-maximization (EM) algorithm is ~43%. (**b**) Dot plot showing freezing levels of animals presented with the New 7 kHz tone. Rats are plotted in blue (discriminators) and red (generalizers). Grey dashed line represents the threshold (43%) generated by the EM-GMM algorithm.

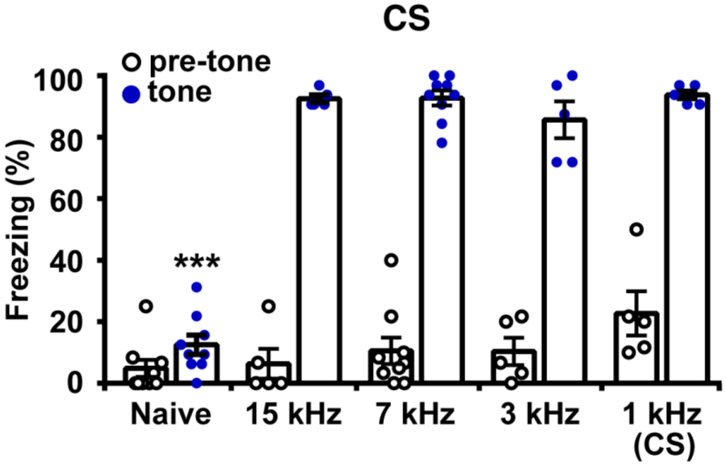

Supplementary Figure 3. Freezing response to CS in each behavioral group. There were significant differences between groups in freezing response to the conditioned stimulus (CS) ($F_{(4, 28)}$ = 149.50, P < 0.001). *Post-hoc* multiple comparisons revealed that naïve animals were significantly lower that 15 kHz, 7kHz, 3 kHz and 1 kHz groups (P < 0.001 in each comparison), while there were no differences among all other conditions (P > 0.05 in each comparison). One-way ANOVA with Newman-Keuls *post-hoc* tests. All data are mean and SEM.

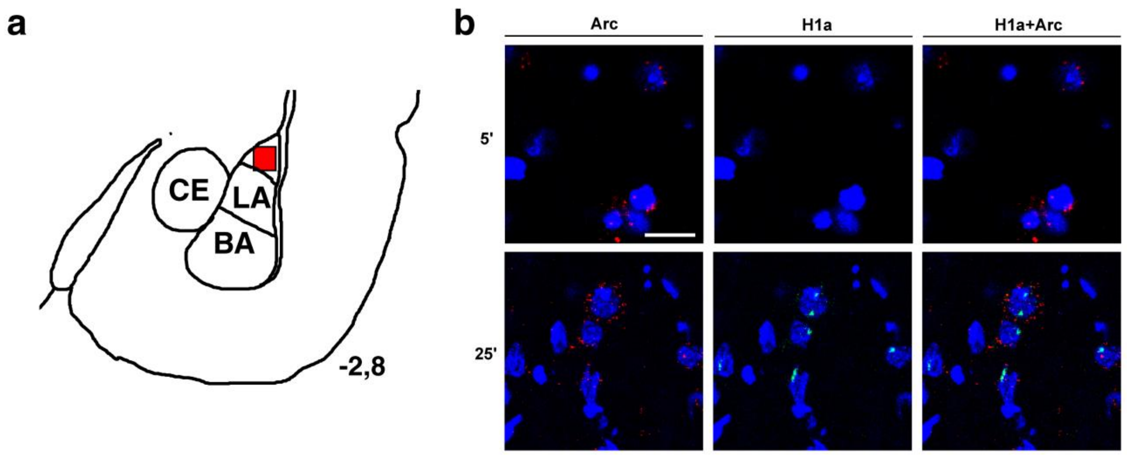

Supplementary Figure 4. Temporal profiles of Arc and H1a RNA expression detected using catFISH. a) A catFISH analysis was performed in the lateral nucleus of the amygdala (LA). The section diagram was drawn on the basis of our DAPI-stained sections. **b**) Time-dependent expression of *Arc* and *H1a* mRNA following CS presentation in the LA. Rats were sacrificed immediately or 22 minutes after the behavioral test. Immediately after the behavioral procedure, *Arc* was expressed in nucleus, whereas *H1a* expression appeared in the nucleus only 25 minutes after the session. At this time point, *Arc* was already detectable in the cytoplasm. Scale bar, 20 µm.

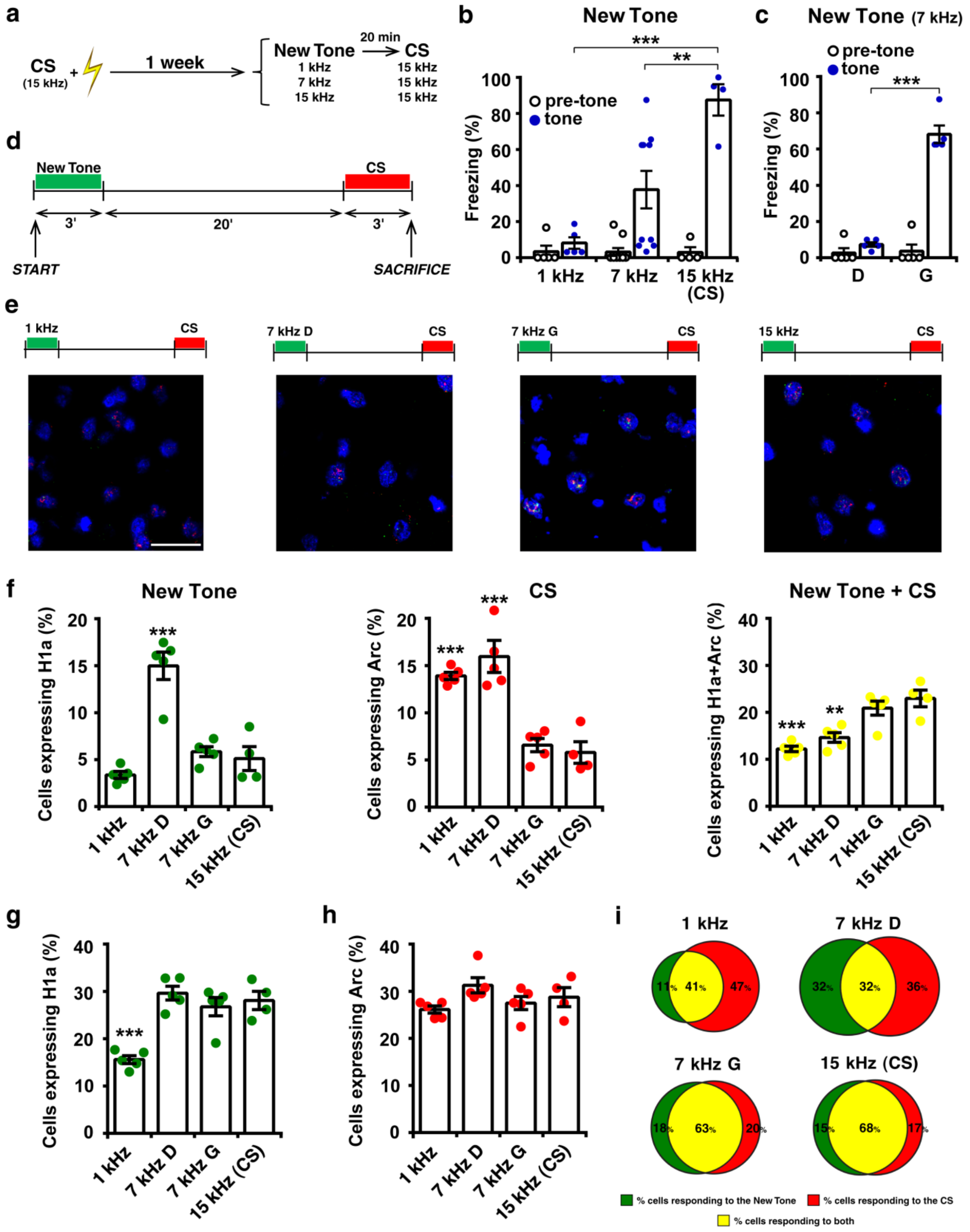

Supplementary Figure 5. catFISH analysis of LA activity in animals in which the frequency of tones was counterbalanced. a) Experimental design of behavioral and catFISH experiments. Training was performed by using 15 kHz tone as CS. One week

after training, rats were presented with a new tone that was different in each behavioral group (i.e., 1 kHz, *n* = 5; 7 kHz, *n* = 10 and 1 kHz, *n* = 4). After 20 min, they were exposed to the CS (15 kHz). (**b**) There were significant differences between groups in freezing response during the presentation of tones of different frequencies ($F_{(2, 16)}$ = 10.42, P < 0.01). Freezing levels of animals exposed to the 15 kHz tone (CS) were significantly higher than 7 kHz (P < 0.01) and 1 kHz conditions (P < 0.001). (**c**) Freezing in "discriminator" animals (D, *n* = 5) was lower than that observed in "generalizer" (G, *n* = 5) animals during the 7 kHz tone delivery ($t_{(8)}$= 12.07, P < 0.001). (**d**) Time course of catFISH experiments. (**e**) Representative images showing neurons expressing single nuclear *H1a* (green arrows) and *Arc* (red) mRNA and double-labeled cells (yellow) in the 1 kHz, 7 kHz (discriminators and generalizers) and 1 kHz groups. Scale bar, 20 µm. (**f**) In the "discriminator" group, dot-plots graphs showed an increase in *H1a*- (new tone) expressing neurons with respect to the other conditions (P < 0.001). Both 7 kHz discriminators and 1 kHz groups showed an increase in *Arc*- (CS) expressing neurons with respect to 7 kHz generalizers and 15 kHz (CS) group (P < 0.001) and a decrease in double-labeled cells in comparison to 7 kHz generalizers and 15 kHz (CS) group (1 kHz: P < 0.001; 7 kHz D: P < 0.01). (One-way ANOVA: $F_{(3, 15)}$ = 28.63, P < 0.001 (*left*); $F_{(3, 15)}$ = 21.59, P < 0.001 (*middle*); $F_{(3, 15)}$ = 16.18, P < 0.001 (*right*)). (**g**) The total rate of *H1a* was lower in the 1 kHz group in comparison to other groups ($F_{(3, 15)}$ = 17.09, P < 0.001 in each comparison). (**h**) The total rate of *Arc* was high and similar between groups ($F_{(3, 15)}$ = 2.35, P > 0.05). (**i**) Scaled Venn diagrams showing the percentage of *H1a*- (green), *Arc*- (red) and *H1a*+*Arc*- (yellow) labeled cells in the different experimental conditions. In 7 kHz D group the neuronal populations activated during both new tone or

CS presentation were less overlapped with respect to other groups. * $P < 0.05$, ** $P < 0.01$, *** $P < 0.001$. All data are mean and SEM. One-way ANOVA with Newman-Keuls test [(**b**), (**f**), (**g**), (**h**)]; unpaired *t* test (**c**).

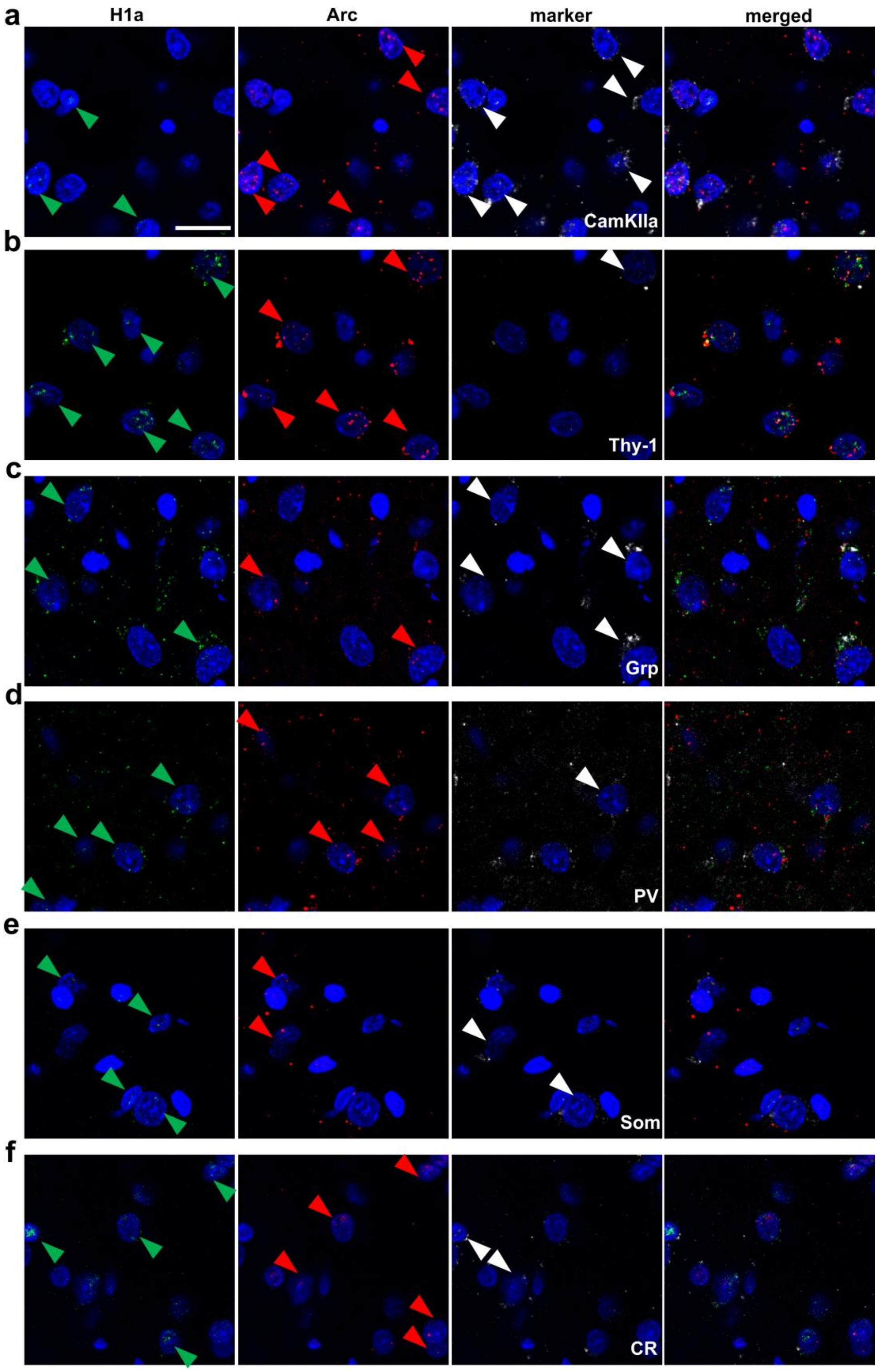

Supplementary Figure 6. Representative images of triple catfish analysis performed in showing discriminator rats following a new tone or CS presentation within the LA. (a-f) Additional representative photomicrographs of triple catfFISH

showing neurons expressing *H1a (*green arrows*)*, *Arc* (red arrows) and *CamKIIa* (**a**), *Thy-1* (**b**), *Grp* (**c**), *PV* (**d**), *Som* (**e**) and *CR* (**f**) (white arrows). Each image was acquired in a different field with respect to regions showed in Fig. 3. Scale bar: 20 µm.

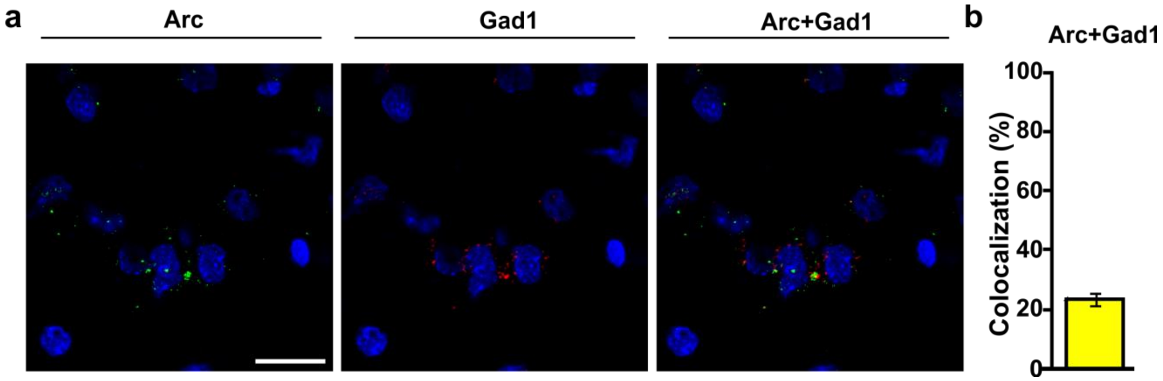

Supplementary Figure 7. In LA, some *Arc*- expressing neurons displayed an inhibitory phenotype. (a) Representative images showing *Arc* and *Gad1* mRNA expression in LA. Scale bar: 20 µm. **(b)** catFISH analysis revealed the expression of *Gad1* in *23.35* ± *2.28*% of LA neurons expressing *Arc*. All data are mean and SEM.

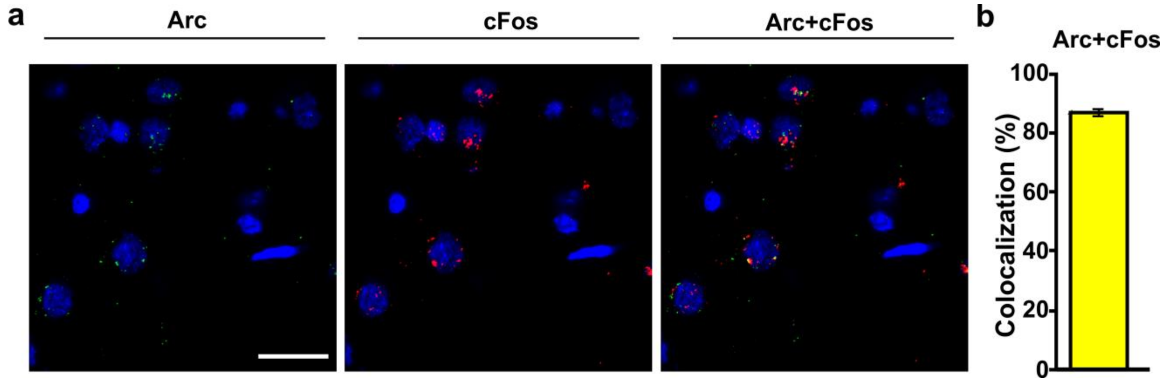

Supplementary Figure 8. In LA, the majority of *Arc*- expressing neurons expressed also *cFos* mRNA. (a) Representative images showing *Arc* and c*Fos* mRNA expression in LA. Scale bar: 20 µm. **(b)** catFISH analysis showed that 87.23% ± *1.25* of *Arc*-labeled neurons expressed also *cFos* mRNA. All data are mean and SEM.

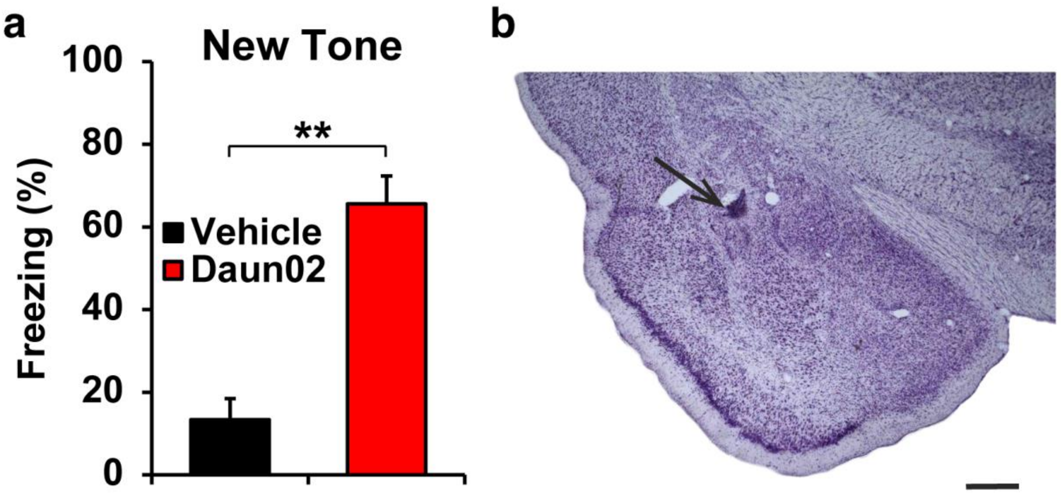

Supplementary Figure 9. Validation of Daun02 experiments. (**a**) Freezing responses during new tone presentation were significantly higher in Daun02-injected rats was significantly higher than in Vehicle-injected animals ($t_{(4)}$ = -6.17, P < 0.01). (**b**) Representative micrographs of Nissl-stained tissues obtained from animals injected with Daun02 within the LA. The arrow indicates the position of the needle track. Scale bar, 500 µm. ** P < 0.01; unpaired *t* test (**a**). All data are mean and SEM.

Group	*n*	Mean Predicted Reactivation Ratio	Mean Observed Reactivation Ratio	t	*P*
15 kHz	5	0.30	0.85	18.77	< 0.001
7 kHz D	5	0.27	0.53	6.07	< 0.01
7 kHz G	4	0.31	0.78	34.59	< 0.001
3 kHz	5	0.37	0.82	38.09	< 0.001
1 kHz	5	0.32	0.85	18.35	< 0.001

Supplementary Table 1. Comparison between predicted and observed reactivation ratio in each behavioral group. For each behavioral group the observed reactivation ratio is significantly higher than the reactivation ratio predicted by chance (paired *t* test).
